# Case report: Isolated dissection of the left gastric artery: an unusual cause of acute abdominal pain

**DOI:** 10.3389/fcvm.2023.1240853

**Published:** 2023-08-16

**Authors:** V. Frey, R. P. Engelberger, E. Psathas

**Affiliations:** ^1^ Department of Internal Medicine, Angiology Unit, HFR Fribourg – Cantonal Hospital, Fribourg, Switzerland; ^2^ Department of Surgery, Vascular Surgery Unit, HFR Fribourg – Cantonal Hospital, Fribourg, Switzerland

**Keywords:** left gastric artery dissection, visceral artery dissection, contrast-enhanced computed tomography, sudden epigastric pain, conservative treatment

## Abstract

Spontaneous and isolated dissection of the left gastric artery is a rare occurrence, with only a handful of cases reported in the medical literature. Clinical presentation may mimic more common intra-abdominal pathologies; however, it is imperative to identify this condition promptly due to its potential serious consequences. This underscores the importance of maintaining a high level of clinical suspicion and including this pathology in the differential diagnosis of patients presenting with acute abdominal symptoms. Hence, this case report aims to increase awareness among clinicians about the importance of identifying and treating this rare condition promptly. A 69-year-old female experienced severe epigastric pain while attending a yoga class, prompting her admission to the emergency department 24 h later due to the persistence of her symptoms. Following imaging work-up utilizing computed tomography angiography (CTA), she was diagnosed with a dissection of the left gastric artery. Notably, there was no associated aneurysm or any evidence of ischemia in the esophageal or gastric wall. Conservative management, including low-dose aspirin and blood pressure control, was implemented. After 6 months of follow-up, CTA demonstrated expansion of the true lumen and the absence of secondary aneurysm formation, leading to discontinuation of aspirin. The management of spontaneous dissection of visceral arteries is primarily determined by the presence of complications and organ ischemia. In the case of uncomplicated visceral artery dissections, first-line treatment comprises surveillance and antiaggregation. Nevertheless, the optimal duration of antiplatelet therapy and the necessity for long-term follow-up remain unclear. Endovascular or surgical interventions should be reserved for patients exhibiting deteriorating symptoms or complications, and the decision to pursue these interventions should be made on a case-by-case basis.

## 1. Introduction

Dissections of visceral arteries are most frequently secondary to acute dissection of the thoraco-abdominal aorta, in which case, acute thoracic or lower back pain is the predominant symptom. Spontaneous, isolated acute dissection of a splanchnic artery can present with diffuse or localized acute abdominal pain, as well as other less specific symptoms, mimicking other more common intra-abdominal pathologies. Isolated acute dissection of an intra-abdominal splanchnic artery is a very uncommon cause of acute abdominal pain. Yoshimi and al. reported an incidence rate of one per 1,214 abdominal CT scans, and rate of one per 462 in emergency abdominal CT scans (1). The most frequently affected arteries being the superior mesenteric and the celiac artery (2). However, isolated dissections of the left gastric artery (LGA) are extremely rare with only 5 published cases in the literature.

## 2. Case description

**Figure 1:**
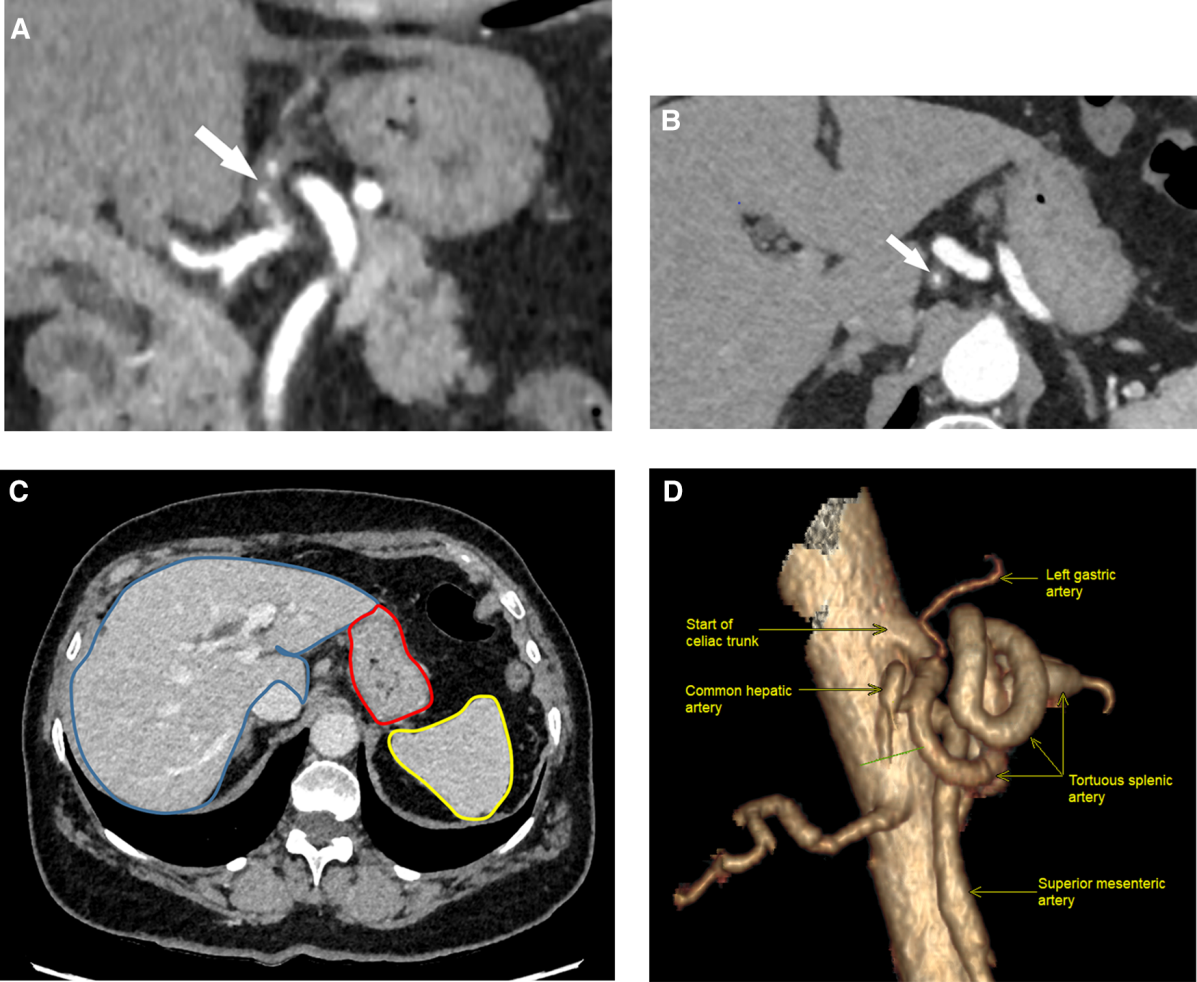
Helicoidal 512 slice computed tomography angiography in arterial phase with 1.3 mm slices. (**A**) Sagittal view showing eccentric wall thickening of the LGA (arrow) from its start of the coeliac artery, evoking a dissection. (**B**) Coronal view of LGA dissection showing a diffuse irregular luminal narrowing (arrow). LGA, left gastric artery. (**C**) Normal perfusion of gastric wall (red mark), liver (blue mark) and spleen (yellow mark) in portal phase. (**D**) 3D-reconstruction of the celiac trunk anatomy (right lateral view, 90°).

A 69-year-old Caucasian female was admitted to the emergency department due to acute epigastric pain that had spontaneously occurred during a yoga session while doing a “bridge stretch” (reclined backbend) and had persisted for the past 24 h. On admission, she reported experiencing epigastric pain and nausea but had not vomited. Her medical history was notable for arterial hypertension and a laparoscopic cholecystectomy that had occurred one year prior. Physical examination revealed upper abdominal tenderness and abdominal guarding upon deep palpation. While vital parameters were within the normal range, blood pressure was elevated at 186/114 mmHg with a normal heart rate of 72 bpm. Laboratory tests indicated slightly elevated CRP levels at 7.4 mg/L (normal range <5) with normal total leucocyte count. Blood pressure was treated with a short-acting calcium antagonist per os. An abdominal ultrasound scan showed no abnormalities, but due to persistent abdominal pain, a computed tomography angiography (CTA) was ordered to rule out mesenteric ischemia. Imaging revealed patency of the celiac trunk and both mesenteric arteries, along with an aberrant left hepatic artery (LHA) originating from the distal part of the LGA. The arterial wall of the LGA from its origin up to the aberrant LHA was thickened, with partial thrombosis of the lumen, indicating isolated arterial dissection. No associated aneurysm or signs of organ malperfusion were observed (Figures 1A–D).

**Figure 2:**
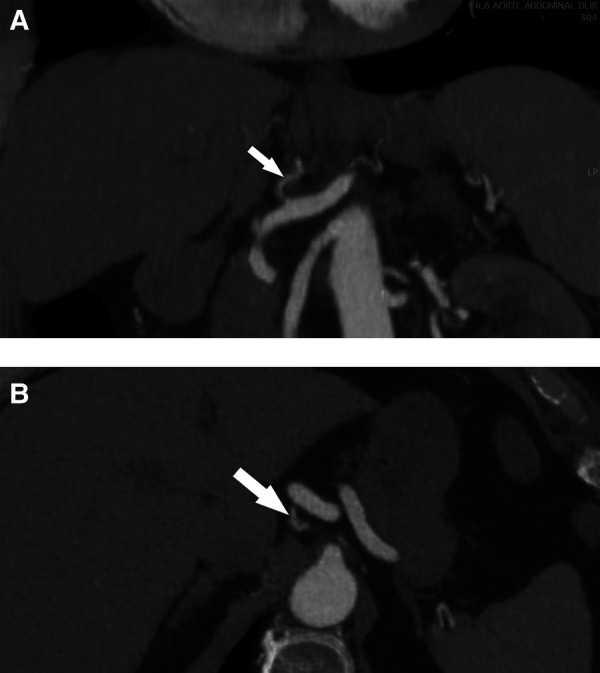
Helicoidal 512 slice computed tomography angiography at 6 months in arterial phase with 1.3 mm slices. (**A**) Sagittal view of a less-thickened LGA (arrow) with near complete resolution of dissection. (**B**) Coronal view with a recanalization of the lumen of the LGA (arrow). LGA, left gastric artery.

Based on these findings, conservative management was proposed, consisting of blood pressure control and close monitoring. The patient had no postprandial pain and was advised to eat normally as tolerated. Single antiplatelet therapy with aspirin at a dosage of 100 mg daily was initiated, and the patient was followed in an outpatient setting. Symptoms gradually improved, and the patient became asymptomatic after 7 days. A follow-up CTA at 6 months showed expansion of the true lumen of the LGA, and no secondary aneurysm formation or imaging signs in favor of fibromuscular dysplasia in other vascular beds were observed (Figures 2A,B). Based on these imaging findings and the absence of recurrent symptoms, antiplatelet therapy was discontinued following a multidisciplinary panel discussion.

## 3. Discussion

Isolated splanchnic artery dissections represent an uncommon cause of acute abdominal pain. Most of them involve the celiac trunk or the superior mesenteric artery (SMA), and in the absence of organ malperfusion, conservative treatment with either antiplatelet agents or therapeutic anticoagulation is associated with favorable outcomes and is a safe first-line treatment (3, 4).

The few cases of isolated dissections of smaller splanchnic arteries, without involvement of the celiac or superior mesenteric artery reported in the literature are summarized in Table 1, with 5 of these case reporting dissections of the LGA. Isolated dissection of the LGA without an associated aneurysm, as in our case, is even more uncommon (5–7).

**Table 1:** Characteristics of included studies.

Study	Year	Involved arteries	Associated aneurysm	Treatment	Follow-up
Ichiba (8)	2016	LGA	Yes	Embolization^a^	CTA^b^ (3 & 6 months)
Kwon (5)	2016	LGA	No	Conservative^b^	Clinical (7 days)
Lim (9)	2016	RGA, LHA	Yes	Embolization	–^b^
Tago (6)	2017	LGA	No	Conservative^b^	CTA^b^ (6 days)
Santos (7)	2019	LGA	No	Anticoagulant/Antiplatelet	Yes ^(b)^
D'Souza (10)	2019	LGA	Yes	Antiplatelet	CTA ^(b)^
Our case	2023	LGA	No	Antiplatelet	CTA

LGA, left gastric artery; RGA, right gastric artery; LHA, left hepatic artery; CTA, computed tomography angiography.

^a^ With microcoil and N-butyl-2-cyanoacrylate.

^b^ Not specified.

Kwon et al. (5) reported a case of a 77-year-old woman presenting with epigastric pain and imaging findings of dissection of the left gastric artery (LGA) without an aneurysm on CTA. A similar anatomical variation was found where the left hepatic artery (LHA) was arising from the LGA. Symptoms resolved after one week with conservative treatment, although the authors provided no further details on the exact medical treatment and follow-up.

Tago et al. (6) reported a case of a 52-year-old man with sudden epigastric pain after eating, in whom a spontaneous dissection of the LGA without an aneurysm was confirmed with CTA. In this case, the LGA was arising directly from the abdominal aorta. The patient was treated conservatively, with no further details regarding type and duration of the treatment, although expansion of the true lumen was observed on follow-up CTA after 6 days.

Santos et al. (7) reported on a 44-year-old man with a 12-hour history of epigastric pain and unremarkable laboratory. Investigations were followed by an upper gastrointestinal endoscopy which showed signs of mild non-erosive distal esophagitis and moderate erosive antral gastritis. Due to ongoing pain, the patient underwent abdominal CTA, which showed an eccentric thickening suggestive of false lumen thrombosis with diffuse irregular thickening of the LGA, suggesting a spontaneous dissection of the LGA. No aneurysm or relevant anatomical variations were found. The patient was treated with anti-coagulation and an antiplatelet agent following a multidisciplinary discussion and had a favorable outcome.

In three further reports, the dissection was associated with an aneurysm and embolization was performed in two cases, for persistent symptoms. Ichiba et al. (8) described a case of a 51 year old woman with acute abdominal pain and nausea. CTA showed a focal LGA dissection and pseudoaneurysm of the distal branch treated conservatively initially with no regression of symptoms after 6 days. Therefore, embolization was erformed with immediate resolution of symptoms.

Lim et al. (9) reported a case of a 51 year old woman with neurofibromatosis which presented with epigastric pain with a CTA showing a LHA pseudoaneurysm with an acute hematoma due to its rupture. Following angiography revealed LHA aneurysm and right gastric artery dissection, which both were embolized.

D'Souza et al. (10) reported a case of a 79 year old men on warfarin for atrial fibrillation which presented in hemorrhagic shock 2 days after a boat incident. Imaging revealed pseudoaneurysms of the LGA measuring up to 9 mm but without signs for active bleeding. Follow-up CTA revealed unchanged pseudoaneurysms but a focal dissection of the LGA with intramural thrombus. Patient was treated with aspirin once daily with favorable outcome.

In our case, the patient reported a sudden pain during a yoga course while doing a “bridge stretch” (reclined backbend), with gradual pain decrease over the next days. The decision to conservatively treat and closely follow-up this patient was based on the absence of an associated aneurysm, thromboembolic or ischemic complications, or coexisting aortic pathology and on the relatively mild clinical presentation with symptoms that showed improvement after the acute episode.

The etiology of isolated LGA dissection remains unclear. We did not find any other imaging signs of fibromuscular dysplasia in the CTA, which would justify long term antiplatelet therapy (11). The most likely etiology would be segmental arterial mediolysis, a non-inflammatory non-atherosclerotic disease typically affecting abdominal visceral arteries. It remains unclear whether the presence of anatomic variations involving the LGA can predispose to an isolated dissection.

**Figure 3:**
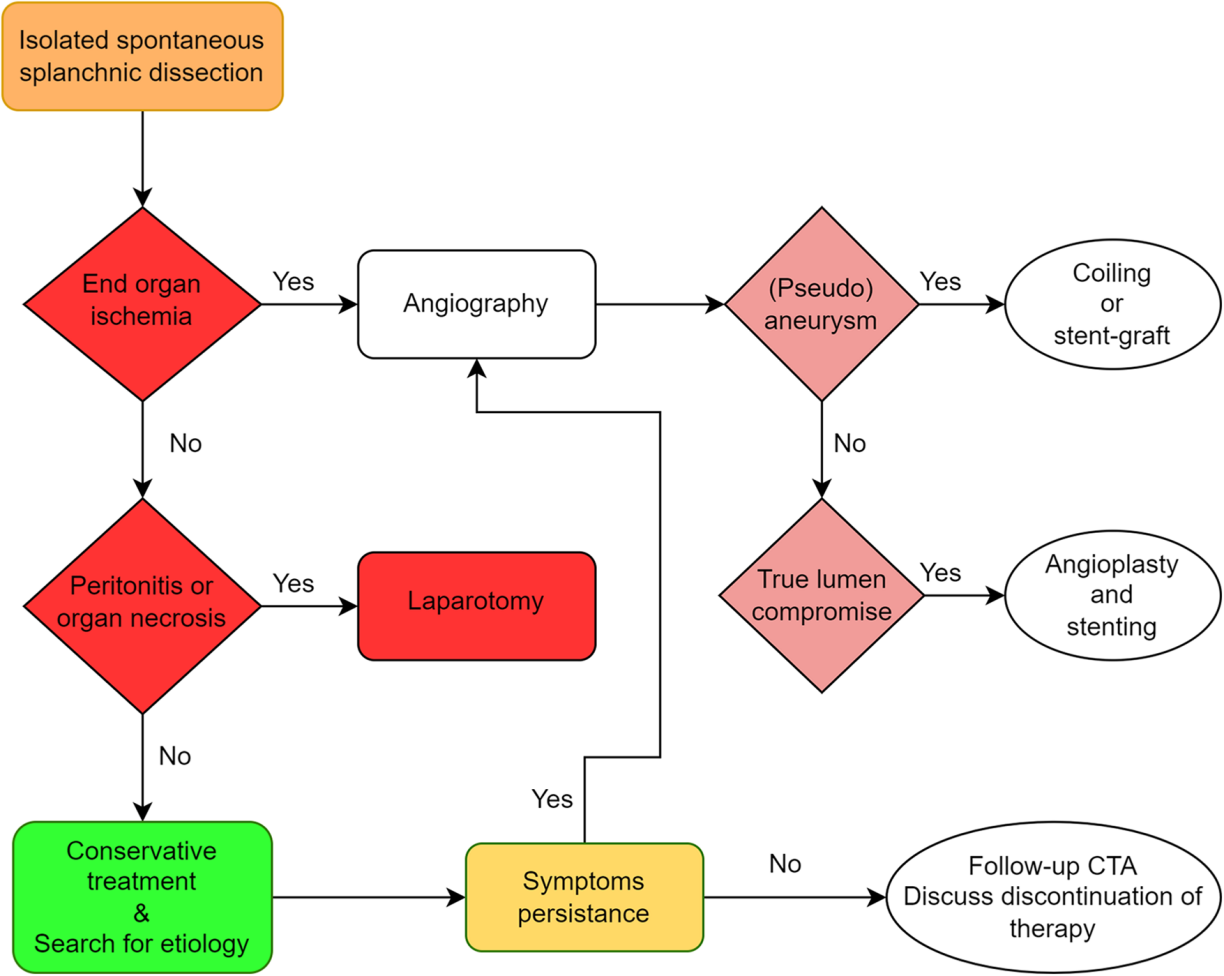
Proposed algorithm for treatment of isolated splanchnic artery dissection.

Overall, anticoagulation or antiplatelet agent and follow-up imaging can be proposed as first line treatment for uncomplicated dissections. The choice between anticoagulation or antiplatelet agents should be based on an individual basis by assessing hemorrhagic risks. With moderate to high bleeding risk, an antiplatelet agent should be preferred over anticoagulation. Endovascular treatment, when feasible, is indicated in case of persistent symptoms, progression of dissection, eminent luminal thrombosis or increasing aneurysmal dilatation, with open surgery with as the last resort in cases of complications or failure of endovascular treatment (10, 12) (Figure 3).

## 4. Patient perspective and conclusion

The patient therefore benefited from a simple, non-invasive treatment and outpatient follow-up, with rapid pain relief, which she particularly appreciated.

Spontaneous isolated LGA dissection is a rare variant of isolated visceral artery dissections and without ischemic or local arterial complications can safely be managed conservatively with antiplatelet therapy or anticoagulation, blood pressure control and clinical surveillance. However, there is no consensus on the optimal frequency and duration of follow-up imaging and medical therapy. Initial surveillance is recommended to monitor for disease progression, which can lead to late complications. In cases where patients experience instability or worsening symptoms despite aggressive medical management, endovascular intervention should be considered. Open surgery is reserved for those patients with peritonitis or failed endovascular treatment.

## Data Availability

The original contributions presented in the study are included in the article, further inquiries can be directed to the corresponding author.
